# Risk of low birthweight associated with the timing and frequency of antenatal care visits in Lao PDR: a retrospective cohort study

**DOI:** 10.1186/s12884-023-05442-7

**Published:** 2023-02-17

**Authors:** Shiori Nagatani, Sayaka Horiuchi, Kenzo Takahashi, Masaaki Matsuura, Kongsit Ounchit, Kazue Yamaoka

**Affiliations:** 1 Project for Sustainable Development and Quality Assurance of Healthcare Professionals, Japan International Cooperation Agency, Lao PDR Teikyo University Graduate School of Public Health, 2-11-1 Kaga, Itabashi, Tokyo Japan; 2 Center for Birth Cohort Studies, University of Yamanashi, Shimokato, Chuo, Yamanashi, 1110 Japan; 3 Teikyo University Graduate School of Public Health, 2-11-1 Kaga, Itabashi, Tokyo Japan; 4 Salavan, Lao People’s Democratic Republic, Salavan Provincial Hospital, Phonkeo Village, Salavan District, Salavan Province, Lao People’s Democratic Republic

**Keywords:** Antenatal care, Low birthweight, Timing of care, Health care utilisation, Socioeconomic status

## Abstract

**Background:**

Antenatal care (ANC) plays an important role in preventing low birthweight (LBW). Whereas the government of Lao People’s Democratic Republic (Lao PDR) has committed to increasing the usage of ANC, little attention has been given to the early initiation of ANC. The present study assessed the influence of delayed and fewer ANC visits on LBW in the country.

**Methods:**

This is a retrospective cohort study conducted at Salavan Provincial Hospital. Study participants were all pregnant women who gave birth at the hospital between 1 August 2016 and 31 July 31 2017. Data were collected from medical records. Logistic regression analyses were performed to quantify the relationship between ANC visits and LBW. We also investigated factors associated with inadequate ANC visits: first ANC visit after the first trimester or < 4 ANC visits.

**Results:**

The mean birth weight was 2808.7 g [standard deviation: SD 455.6]. Among 1804 participants, 350 (19.4%) had babies with LBW, and 147 (8.2%) had inadequate ANC visits. In multivariate analyses, compared to participants with adequate ANC visits, those with ≥ 4 ANC visits and the first ANC visit after the second trimester, those with < 4 ANC visits, and those with no ANC visits had higher odds ratios (ORs) of LBW: 3.77 (95% confidence interval: CI = 1.66–8.57), 2.39 (95% CI = 1.18–4.83) and 2.22 (95% CI = 1.08–4.56), respectively. Younger maternal age (OR 1.42; 95% CI = 1.07–1.89), government subsidisation (OR 2.69; 95% CI = 1.97–3.68) and ethnic minority (OR 1.88; 95% CI = 1.50–2.34) were associated with increased risk of insufficient number of ANC visits after adjusting for covariates.

**Conclusions:**

Frequent and early initiation of ANC was associated with a reduction in LBW in Lao PDR. Encouraging childbearing-aged women to receive sufficient ANC at proper timing may lead to a reduction in LBW and improvement in short- and long-term health outcomes of neonates. Special attention will be needed for ethnic minorities and women in lower socioeconomic classes.

**Supplementary Information:**

The online version contains supplementary material available at 10.1186/s12884-023-05442-7.

## Background

Low birthweight (LBW) refers to weight at birth of less than 2500 g, regardless of gestational age [1]. Approximately 20.5 million neonates worldwide, equivalent to one in every seven, were estimated to have LBW in 2015 [1, 2]. Epidemiological observations showed that LBW neonates were at greater risk of dying than non-LBW neonates during the neonatal period, owing to birth asphyxia and infection [3]. It was reported that LBW neonates born early accounted for approximately 50% of neonatal deaths worldwide. LBW also involves subsequent adverse outcomes. Neonates born small are more likely to develop chronic conditions later in life, including obesity, type 2 diabetes, hypertension, cardiovascular and respiratory diseases, as well as cognitive impairment [4–7]. The influence of LBW can also persist into the next generation as girls born small have a greater risk of birthing small babies later in life [8].

The prevalence of LBW varies across countries and regions. Approximately 90% of all LBW neonates are born in low- and middle-income countries, and more than half are born in Asia [2]. Given the range of the adverse consequences of LBW, reducing LBW has been a public health priority. The World Health Assembly endorsed *The Global Nutrition Target,* which established a commitment to reducing LBW by 30% in 2012 [9]. However, progress in reducing LBW has been stagnant, with the global annual average rate of reduction in LBW at only 1.23% in the period from 2000 to 2015 [2].

Several factors are known to affect birthweight. Premature birth, that is, birth before 37 weeks of gestation, is a major risk factor for LBW [10]. Another important factor is foetal growth restriction (FGR), a condition in which a foetus is unable to achieve its potential size because of a birth defect, maternal advanced age, maternal consumption of alcohol, tobacco and/or drugs or pregnancy-related complications [1, 10–14]. Deprived maternal socio-economic conditions are also associated with a higher risk of LBW [15].

To address these risk factors and improve maternal and foetal health, provision of appropriate care throughout pregnancy is crucial [16]. Antenatal care (ANC) plays an important role in providing critical health care services, including risk identification, disease prevention and health promotion. Nonetheless, mothers in low- and middle-income countries often have limited access to evidence-based, high-quality care in a timely manner, especially in rural areas or in settings of conflict [17, 18]. Four-time-focused ANC (FANC), which refers to at least 4 ANC visits during pregnancy starting before the 12^th^ week of pregnancy, was recommended by the World Health Organization (WHO) in 2007 [19]. Although the WHO’s new guidelines on ANC (2016) increased the optimal frequency of ANC visits from four to eight [20], FANC remains a target in resource-limited settings where the prevalence of ANC remains low.

Following the WHO recommendation in 2007, the Ministry of Health in Lao People's Democratic Republic (Lao PDR) set a target to increase the percentage of women who received ANC four times or more (ANC4 +) to 80% by 2025 [21]. Pregnant women were recommended to visit either a health centre or the district/provincial/central hospital to receive ANC at least once both in the first and second trimesters and twice in the third trimester [22]. With this governmental emphasis, the national ANC4 + coverage in Lao PDR increased from 36.9% in 2012 to 62.2% in 2017 [23, 24]. However, the ANC4 + coverage in Salavan province showed little progress in the same period (23.2% in 2012, 35.9% in 2017) [23, 24]. Even worse, only 32.9% of the first ANC visit occurred during the first trimester of pregnancy in Salavan province [24]. Thus, there have been concerns that interventions through ANC may not work sufficiently to prevent LBW for pregnant women with delayed initiation of ANC and fewer visits. A Cochrane review reported that a reduced number of ANC visits might increase the risk of perinatal mortality, compared to the standard number of visits in low-resource settings where the standard number is already low [25]. A case–control study in Lao PDR showed that LBW was more likely to occur in a group experiencing less than four ANC visits [26]. Although the importance of early initiation of ANC has been recognised by WHO [20], the influence of delay in the first ANC visit on birth outcomes has not been fully evaluated in Lao PDR. The present study aims to quantify the combined influence of the frequency and timing of ANC visits on the risk of LBW and assess the factors that contribute to reduced and delayed ANC. Findings of the present study will provide useful suggestions to policymakers in Lao PDR and other resource-limited settings for improving the ANC program.

## Methods

### Study design and setting

In this retrospective cohort study, a survey of patients’ records was conducted at Salavan Provincial Hospital in Lao PDR in 2017. Salavan Provincial Hospital is the largest hospital in Salavan province, serving as a secondary referral hospital in the country. Salavan province is in the southern part of Lao PDR and was reported to be one of the poorest provinces in that country [27]. Lao PDR is ethnically diverse, with the Lao being the largest ethnic group, accounting for almost half of the Salavan population (48.8%). Other ethnic minorities have cultures and languages different from Lao. Buddhists account for the majority (69.3%) in the province. The prevalence of LBW in the province was 11.7% in 2017, which was higher than the national average of 9.7% [24]. According to the national strategy, Salavan province promotes ANC to be provided at least four times during pregnancy. In addition, physical examinations are performed to detect danger signs, health education is offered and iron–folic-acid tablets are distributed. The service package of ANC in Salavan province as of 2017 is summarised in Table 1.

**Table 1:** Service package at antenatal care in Salavan province, Lao PDR

Timing	Service contents
First visit	-Estimate the date of confinement
	-Measure weight, height, blood pressure and symphysis-fundal height
	-Check foetal heartbeat by stethoscope according to gestational week
	-Provide a maternal and child health (MCH) handbook
	-Administer HIV testing and counselling
	-Furnish tetanus toxoid vaccination depending on immunisation history
	-Provide counselling on next visit
Subsequent visits	-Determine weight gain and symphysis-fundal height
	-Evaluate blood pressure
	-Check foetal heartbeat
	-Provide dietary counselling and iron–folic-acid tablets
	-Record MCH handbook

### Study participants

Study participants were pregnant women who gave birth at Salavan Provincial Hospital between 1 August 2016 and 31 July 2017. Women who experienced stillbirths, lived outside the province, or had foreign nationality were excluded from our study. The required sample size was 1150 to detect a 1.5-fold increase in odds of LBW among participants with adequate frequency and timing of ANC visits, compared to participants with reduced or delayed ANC; the statistical results had 80% power and a 5% significance level.

### Data collection

Data on all variables were collected from Obstetric and Outpatient Register Records stored in the Department of Obstetrics–Gynaecology and in the Department of Maternal and Child Health at Salavan Provincial Hospital. Records were completed at delivery and at every ANC visit by health workers at Salavan Provincial Hospital. All data were anonymised at data entry.

### Primary outcome

The primary outcome was LBW, defined as birthweight of less than 2500 g. Birthweight was measured with a scale on the day of birth by skilled birth attendants (SBA). The SBA, defined as a midwife, nurse, physician or medical assistant with midwifery skills including a minimum capacity to provide new-born care [28], followed standard procedures for weighing.

### Exposure

The exposure was set as the adequacy of ANC. Adequacy was classified into the following four groups according to the frequency of ANC visits and timing of the first ANC visit: Class 0 (total ANC visits ≥ 4 and first visit in the first trimester), Class 1 (total ANC visits ≥ 4 and first visit in the second or third trimester), Class 2 (total ANC visits < 4) and Class 3 (no ANC throughout the pregnancy). The frequency of ANC visits and timing of the first visit were confirmed when a pregnant woman visited the hospital to give birth by checking her maternal and child health handbook or by interview if she presented without a handbook. From this recording, the frequency of ANC visits was classified as ≥ 4 times (4 +), 3 times, twice, once and never. The timing of the first ANC visit was classified as either the first (before 12 gestational weeks), second (13 to 28 weeks) or third (after 29 weeks) trimester.

### Other covariates

Maternal age at birth, season of birth (rainy season or dry season), mode of delivery (vaginal or caesarean birth), residential area (provincial capital or other districts), reproductive history, type of health insurance and ethnicity were all considered as potential confounders in the analyses. Reproductive history was classified into primipara (no previous birth) or multipara (previous birth), as primiparas were reported to be associated with a higher risk of having an LBW neonate [29]. The socio-economic status of mothers has been reported as an important confounding factor; however, it was difficult to obtain household income from hospital records. Instead, we used information on types of health insurance as a proxy for socio-economic status of the household, because the type of health insurance prior to the implementation of national health insurance was dependent on socio-economic status: the poor were covered by a different financial scheme than those who worked in formal and informal sectors [30]. We categorised the health insurance schemes into two categories. One is for employees in the formal sector, such as civil servants, or in the informal sectors and their spouses. The other is a government subsidisation scheme for pregnant women and the poor [31]. Maternal ethnic group was considered to be related to economic status and lifestyles that could influence health-seeking behaviour and health outcomes.

### Data analysis

First, distributions of birthweight, frequency of ANC visits, timing of the first ANC visit and other covariates across the study participants were examined and summarised according to mean [standard deviation: SD] or number (%) as appropriate. Then, a univariate logistic regression analysis was applied to investigate the risk of LBW in relation to each of the explanatory variables and other covariates. Next, a multivariate logistic regression analysis was performed to evaluate the association between LBW and the adequacy of ANC visits, adjusted for other variables that were statistically associated with LBW in the univariate analysis. Participants with missing data for any variable were excluded from the multivariate analysis; however, sensitivity analyses were performed by imputing missing data 30 times using the chained equation and comparing the results with those of analyses with complete data. Additionally, we investigated factors associated with an insufficient number of ANC visits and delay in the first ANC visit, separately, using logistic regression analysis.

A significance level of 5% with the two-tailed test was applied for all significance tests. All the analyses were performed with Stata/MP 16.0.

## Results

**Fig. 1:**
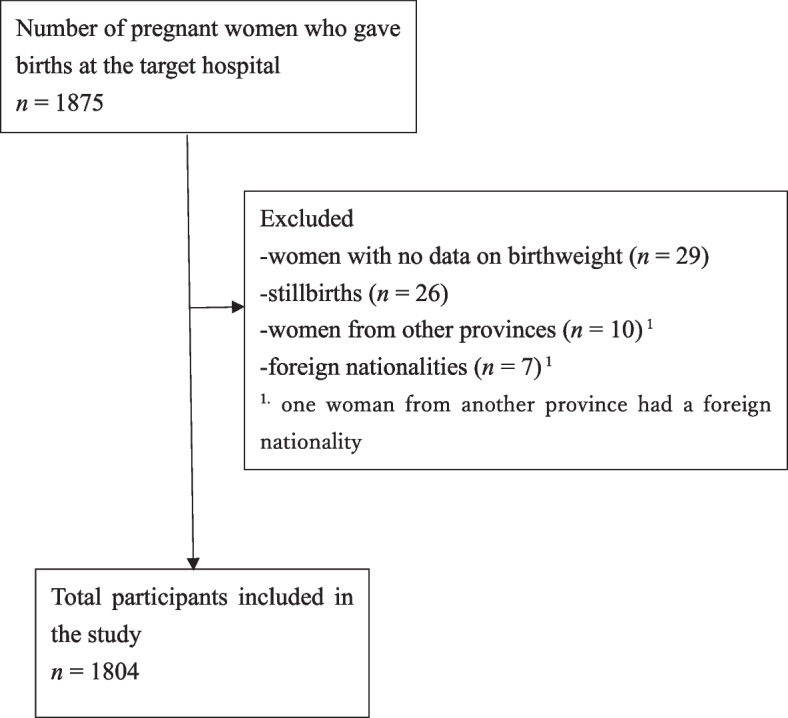
Selection of study participants

During the period from 1 August 2016 to 31 July 2017, 1875 pregnant women gave birth in Salavan Provincial Hospital (Fig. 1). Of these, 29 did not have birthweight records, 26 experienced stillbirths, 10 were from other provinces and 7 were of foreign nationalities (one woman from another province had a foreign nationality); these were excluded from the analysis. Ultimately, 1804 participants were included in the present study.

Table 2 shows the mean birthweight, frequency of LBW and adequacy of ANC visits, as well as the background characteristics of the participants. Among all participants, 905 (50.2%) women had ANC 4 + , whereas 135 (7.5%) never had ANC. Only 166 (9.2%) had the first ANC visit in the first trimester of pregnancy. Adequate ANC visits, that is, the first ANC in first trimester and ANC 4 + (Class 0), were made by 147 (8.2%) participants.

**Table 2:** Background characteristics and outcomes of study participants (*N* = 1804)

Variables	Frequency (%) or mean [SD]
**Neonate birthweight (g)** mean [SD]	2808.7 [455.6]
≧2500	1,454 (80.6)
< 2500 (LBW)	350 (19.4)
**Number of ANC visits (time)** mean [SD]	3.6 [2.0]
4 +	905 (50.2)
3	329 (18.2)
2	296 (16.4)
1	126 (7.0)
Never	135 (7.5)
Missing data	13 (0.7)
**Timing of first ANC visit (weeks)** mean [SD]	20.1 [7.0]
≦12 (First trimester)	166 (9.2)
12 < ≦28 (Second trimester)	870 (48.2)
> 28 (Third trimester)	185 (10.3)
Never visited	135 (7.5)
Missing data	448 (24.8)
**Adequacy of ANC visits**	
Class 0 (total ANC ≥ 4 AND first ANC in first trimester)	147 (8.2)
Class 1 (total ANC ≥ 4 AND first ANC in second or third trimester)	534 (29.6)
Class 2 (total ANC < 4)	751 (41.6)
Class 3 (never visited ANC throughout the pregnancy)	135 (7.5)
Missing data	237 (13.1)
**Delivery season**	
Rainy season	829 (46.0)
Dry season	975 (54.0)
**Mode of delivery**	
Caesarean	309 (17.1)
Vaginal	1479 (82.0)
Missing data	16 (0.9)
**Maternal age (year)** mean [SD]	25.4 [5.6]
≧20	1509 (83.6)
< 20	276 (15.3)
Missing data	19 (1.1)
**Type of health insurance**	
Formal and informal sector	237 (13.1)
Government subsidisation	1564 (86.7)
Missing data	3 (0.2)
**Residential area**	
Salavan district	1376 (76.3)
Other districts	425 (23.6)
Missing data	3 (0.1)
**Ethnicity**	
Lao	1245 (69.0)
Minority	552 (30.6)
Missing data	7 (0.4)
**Religion**	
Buddhist	1250 (69.3)
Animist or Christian	547 (30.3)
Missing data	7 (0.4)
**Parity**	
Multipara (≧1)	1051 (58.3)
Primipara (= 0)	746 (41.4)
Missing data	7 (0.3)
**Neonatal sex**	
Male	958 (53.1)
Female	808 (44.8)
Missing data	38 (2.1)
**Multiple birth**	
Singleton	1763 (97.7)
Twin	41 (2.3)
**Gestational week of delivery**	
Full-term birth	1693 (93.8)
Premature birth	107 (5.9)
Missing data	4 (0.3)

Numbers are number (%) unless otherwise specified

*ANC* Antenatal care, *SD* Standard deviation, *LBW* Low birthweight

In the univariate analyses, adequacy of ANC visits was inversely associated with LBW (Supplementary Table 1). Compared to participants who had adequate ANC visits (Class 0), the odds of having an LBW neonate was approximately twice that for those with a total ANC visits ≥ 4 and the first visit in the second or third trimester (Class 1) (OR 1.76, 95% CI = 0.95–3.27), greater than a factor of three for those with ANC visits < 4 (Class 2) (OR 3.30, 95% CI = 1.82–5.97) and greater than a factor of four for women who never had ANC (Class 3) (OR 4.34, 95% CI = 2.20–8.56). Besides adequacy of ANC, older maternal age, living outside of Salavan district (Salavan provincial capital), ethnic minority, primipara, multiple birth and lower gestational age at birth were strongly associated with increased odds of LBW; those variables were included in the multivariate analyses. Although religion was strongly associated with LBW, it was omitted from the multivariate analyses owing to strong correlation with ethnicity. After adjusting for covariates, the ORs of LBW for class 3, class 2 and class 1 were 3.77 (95% CI = 1.66–8.57), 2.39 (95% CI = 1.18–4.83) and 2.22 (95% CI = 1.08–4.56), respectively, compared to class 0. The results were consistent with those of analyses with imputed data; however, the lower limit of the 95% CI for class 1 was below 1 (OR 1.99, 95% CI = 0.98–4.05). The trend test (Wald test) produced a p-value of 0.004.

Supplementary Table 2 shows the potential factors related to an insufficient number of ANC visits and delay in the first ANC visit. In the adjusted analyses, younger maternal age (OR 1.42; 95% CI = 1.07–1.89), government subsidisation (OR 2.69; 95% CI = 1.97–3.68) and ethnic minority (OR 1.88; 95% CI = 1.50–2.34) were associated with increased risk of insufficient number of ANC visits. Primipara was associated with a decreased risk of insufficient number of ANC visits (OR 0.78, 95% CI = 0.63–0.96). None of the factors examined were associated with delay in the first ANC visit in the adjusted analysis.

## Discussion

The presented study demonstrated a reciprocal relationship between the risk of LBW and adequacy of ANC. The risk of LBW decreased with an increase in the number of ANC visits, with the risk being further reduced when the first ANC occurred in the first trimester of pregnancy. This linearity suggested a strong relationship between LBW and adequacy of ANC. Although the relationship between LBW and Class 1 (ANC visits ≥ 4 and first visit in second or third trimester) slightly waned in the multivariate analysis with imputed data, the study suggested that the timing of the first ANC visit might influence the effect of ANC on LBW.

The results highlighted the new insight that not only frequency of ANC visits but also the timing of the first ANC visit is important to improve maternal and neonatal health outcomes. The finding provides useful suggestions to decision makers regarding the design and promotion of ANC services in Lao PDR as well as in other resource-limited settings.

Unlike previous studies that focused on the frequency of ANC visits in the prevention of LBW [25, 26], the present study quantified the effects of a delay in the first ANC visit on the risk of LBW, in addition to the effect of fewer ANC visits. The timing of the first ANC visit would be critical for risk identification, disease prevention and health promotion, ultimately affecting the health outcomes of mothers and babies [32, 33]. With regard to the ANC program in Lao PDR, distributing iron folic acid and providing health education on nutrition may play important roles in preventing adverse health outcomes of neonates including LBW [1, 34]. Considering the impact of delayed first ANC visits, public effort should appeal not only to pregnant women but also to all women of childbearing age in encouraging them to initiate the first ANC at the proper time.

Maternal educational attainment and knowledge may be important determinants of insufficient and late initiation of ANC in low- and middle-income countries [35, 36]. A study implemented in Lao PDR reported that women who perceived ANC as being unnecessary and/or felt embarrassed were less likely to start ANC in the first trimester of pregnancy and were less likely to make ANC visits more than four times [36]. We did not examine maternal educational attainment in the present study; however, ethnic minorities and government subsidisation were associated with an insufficient number of ANC visits, suggesting that lower social status might be an important determinant of frequency of ANC visits. Health promotion and educational intervention may help promote the utilisation of ANC. Interestingly, no factors, including those associated with an insufficient number of ANC visits, were associated with delays in the first ANC visit. Maternal knowledge about birth complications and the importance of ANC were also not considered in the present study. As these factors can be considered to play an important role in maternal decision-making, further research would be necessary to evaluate reasons for delaying the first ANC visit.

### Strengths and limitations of the research

The present study is a retrospective cohort study based on hospital records. Although there were some missing data, results of imputed data were consistent with results of complete data. A reverse causality was a potential concern as pregnant women who suspected FGR tended to receive ANC more frequently in some countries where the quality of ANC is high and maternal care is advanced. However, we considered that this would be less likely in Lao PDR, where the foetus size is not routinely measured.

The present study has some limitations. First, information regarding some important confounders was limited. Well-known risk factors for LBW, which include maternal alcohol consumption, cigarette smoking, drug abuse and the presence of pregnancy-related complications such as hypertensive disorders, were not recorded in the hospital records. Therefore, we could not adjust for these confounders in the analyses. In addition, faulty memory by the mothers regarding the number of ANC visits and timing of the first ANC visit might have caused misclassification of exposures. However, this would be non-differential because the ANC status was recorded before childbirth. This would rather have caused underestimation of the real association between LBW and number of ANC visits or the timing of the first ANC visit. Furthermore, the current results might not be representative of the entire population in Lao PDR as only pregnant women who gave birth at the provincial hospital were included in our study. The use of a health facility in Salavan province occurred in 60.4% of childbirths in 2017 [24]. Thus, 40% of pregnant women do not use a health facility for childbirth and might have a much stronger risk of LBW than those who use a health facility. Finally, this study did not consider the quality of ANC. Lack of access to quality care is now recognised as more critical than non-utilisation of the health system for improved health outcomes in low- and middle-income countries [18]. Women who had adequate ANC visits may have had healthier behaviour and, therefore, better health outcomes than those who did not. Further studies to evaluate the quality of ANC and its influence on health outcomes in Lao PDR are warranted.

## Conclusions

The present study revealed that a combination of lower frequency of ANC visits and delayed initiation of the first ANC increased the risk of LBW in Lao PDR. It also showed that maternal age, residential area, minority status and insurance type were associated with inadequacy of ANC visits. Encouraging childbearing aged women to receive the sufficient number of ANC visits with proper timing may lead to a reduction in LBW and improvement in short- and long-term health outcome of neonates. Special attention will be needed for women living in rural areas, with minority status and having a lower socio-economic position. Further studies will be needed to investigate the factors that influence delays in the first ANC visit.

## Supplementary Information

**Additional file 1:** **Supplementary Table 1.** Crude and adjusted odds ratios of low birthweight in association with adequacy of ANC visits.

**Additional file 2:** **Supplementary Table 2.** Crude and adjusted odds ratios of having inadequate-ANC-related risk factors.

## Data Availability

The datasets generated during and/or analysed during the current study are available from the corresponding author on reasonable request.

## Abbreviations

ANC: Antenatal care
ANC4 +: More than four ANC visits
CI: Confidence interval
FANC: Focused ANC
FGR: Foetal growth retardation
Lao PDR: Lao People’s Democratic Republic
LBW: Low birthweight
OR: Odds ratio
SBA: Skilled birth attendant
SD: Standard deviation
