# Supplementary material for: Risk of low birthweight associated with the timing and frequency of antenatal care visits in Lao PDR: a retrospective cohort study
Source: BMC Pregnancy Childbirth. 2023 Feb 17;23:119. doi: 10.1186/s12884-023-05442-7 (PMC9936643; doi:10.1186/s12884-023-05442-7)
Supplement: Supplementary file 1 — Additional file 1: Supplementary Table 1. Crude and adjusted odds ratios of low birthweight in association with adequacy of ANC visits. [file 12884_2023_5442_MOESM1_ESM.docx]

**Supplementary Table 1. Crude and adjusted odds ratios of low birthweight in association with adequacy of ANC visits**

| **Variable** | **Crude OR (95% CI)** | **Adjusted OR (95% CI)**  **Complete data**  **(N = 1542)** | **Adjusted OR (95% CI)**  **Multiple imputation**  **(N = 1804)** |
| --- | --- | --- | --- |
| **Adequacy of ANC visits** |  |  |  |
| Class 0 (total ANC visits ≥ 4 AND first visit in first trimester) | 1 | 1 | 1 |
| Class 1 (total ANC visits ≥ 4 AND first visit in second or third trimester) | 1.76 (0.95–3.27) | 2.22 (1.08–4.56) | 1.99 (0.98–4.05) |
| Class 2 (total ANC visits < 4) | 3.30 (1.82–5.97) | 2.39 (1.18–4.83) | 2.24 (1.13–4.46) |
| Class 3 (no ANC visits throughout the pregnancy) | 4.34 (2.20–8.56) | 3.77 (1.66–8.57)^a^ | 3.45 (1.55–7.71)^b^ |
| **Delivery season** |  |  |  |
| Rainy season | 1 | - | - |
| Dry season | 0.98 (0.78–1.24) | - | - |
| **Mode of delivery** |  |  |  |
| Caesarean | 1 | - | - |
| Vaginal | 0.96 (0.71–1.30) | - | - |
| **Maternal age** |  |  |  |
| ≧ 20 | 1 | 1 | 1 |
| < 20 | 1.90 (1.42–2.54) | 1.66 (1.12–2.47) | 1.64 (1.13–2.38) |
| **Type of health insurance** |  |  |  |
| Formal and informal sector | 1 | - | - |
| Government subsidisation | 1.35 (0.93–1.95) | - | - |
| **Residential area** |  |  |  |
| Salavan district | 1 | 1 | 1 |
| Other districts | 1.80 (1.39–2.33) | 1.17 (0.79–1.74) | 1.02 (0.72–1.45) |
| **Ethnicity** |  |  |  |
| Lao | 1 | 1 | 1 |
| Minority | 1.82 (1.43–2.32) | 1.33 (0.94–1.87) | 1.15 (0.83–1.58) |
| **Religion** |  |  |  |
| Buddhist | 1 | - | - |
| Animist or Christian | 1.82 (1.43–2.32) | -^c^ | -^c^ |
| **Parity** |  |  |  |
| Multipara | 1 | 1 | 1 |
| Primipara | 1.64 (1.29–2.07) | 1.52 (1.09–2.11) | 1.55 (1.14–2.11) |
| **Neonatal sex** |  |  |  |
| Male | 1 | - | - |
| Female | 1.24 (0.98–1.58) | - | - |
| **Multiple birth** |  |  |  |
| Singleton | 1 | 1 | 1 |
| Twin | 33.22 (12.93–85.34) | 75.63 (17.04–335.64) | 40.35 (14.98–108.70) |
| **Gestational age of birth** |  |  |  |
| Full-term birth | 1 | 1 | 1 |
| Premature birth | 152.92 (55.81–419.01) | 168.81 (52.43–543.51) | 147.66 (53.32–408.93) |

ANC: antenatal care, CI: confidence interval, OR: odds ratio

Logistic regression was used to estimate the effect.

In the multivariate analysis, adjusted for variables that are only statistically significant for association with LBW in the univariate analysis.

^a^ Trend test (Wald test): p-value = 0.004, ^b^ Trend test (Wald test): p-value = 0.003, ^c^ Religion was not adopted, as it has strong multi-collinearity with ethnicity. The adjusted OR and 95% CI were 1.11 (0.79–1.53)
