# Supplementary material for: Risk of low birthweight associated with the timing and frequency of antenatal care visits in Lao PDR: a retrospective cohort study
Source: BMC Pregnancy Childbirth. 2023 Feb 17;23:119. doi: 10.1186/s12884-023-05442-7 (PMC9936643; doi:10.1186/s12884-023-05442-7)
Supplement: Supplementary file 2 — Additional file 2: Supplementary Table 2. Crude and adjusted odds ratios of having inadequate-ANC-related risk factors. [file 12884_2023_5442_MOESM2_ESM.docx]

**Supplementary Table 2. Crude and adjusted odds ratios of having inadequate-ANC-related risk factors**

|  | **Number of ANC visits (Total ANC visits < 4 compared to total ANC visits ≥ 4)** | | **Timing of the first ANC visit (first visit in second or third trimester compared to first visit in first trimester)** | |
| --- | --- | --- | --- | --- |
| **Variable** | **Crude OR (95% CI)** | **Adjusted OR (95% CI)^1^**  **(n = 1757)** | **Crude OR (95% CI)** | **Adjusted OR (95% CI)^a^**  **(n = 1211)** |
| **Delivery season** |  |  |  |  |
| Rainy season | 1 | 1 | 1 | 1 |
| Dry season | 0.98 (0.81–1.18) | 0.96 (0.79–1.17) | 0.94 (0.67–1.30) | 0.91 (0.65–1.26) |
| **Maternal age** |  |  |  |  |
| ≧ 20 | 1 | 1 | 1 | 1 |
| < 20 | 1.53 (1.18–1.98) | 1.42 (1.07–1.89) | 1.42 (0.85–2.39) | 1.46 (0.84–2.52) |
| **Type of health insurance** |  |  |  |  |
| Formal and informal sector | 1 | 1 | 1 | 1 |
| Government subsidisation | 2.91 (2.15–3.94) | 2.69 (1.97–3.68) | 1.30 (0.84–2.00) | 1.29 (0.83–2.01) |
| **Residential area** |  |  |  |  |
| Salavan district | 1 | 1 | 1 | 1 |
| Other districts | 1.16 (0.94–1.45) | 0.93 (0.73–1.18) | 0.86 (0.53–1.39) | 0.81 (0.49–1.34) |
| **Ethnicity** |  |  |  |  |
| Lao | 1 | 1 | 1 | 1 |
| Minority | 1.93 (1.58–2.37) | 1.88 (1.50–2.34) | 1.02 (0.69–1.50) | 1.06 (0.71–1.60) |
| **Parity** |  |  |  |  |
| Multipara | 1 | 1 | 1 | 1 |
| Primipara | 0.91 (0.76–1.10) | 0.78 (0.63–0.96) | 0.91 (0.65–1.27) | 0.83 (0.59–1.18) |

ANC: antenatal care, CI: confidence interval, OR: odds ratio

Logistic regression was used to estimate the effect.

**^a^** Adjusted for all other variables in the table
